# Impact of Obesity and Preoperative Knee Subcutaneous Fat Indices as Predictors of Postoperative Functional Outcomes Following Total Knee Arthroplasty: A Comprehensive Retrospective Review

**DOI:** 10.7759/cureus.83968

**Published:** 2025-05-12

**Authors:** Mohammad Adeel Sajid Khan, Mohammed A Ali, Fatima M Atieh, Shayma M Ali, Ahmed M El-Hagrasy, Hammad N Khan, Ahsan Javaid Butt

**Affiliations:** 1 Orthopedic Surgery, King Hamad University Hospital, Manama, BHR; 2 Medicine, School of Medicine, Royal College of Surgeons in Ireland, Muharraq, BHR

**Keywords:** body mass index, functional outcomes, knee osteoarthritis (koa), subcutaneous fat measurements, total knee arthroplasty (tka)

## Abstract

Introduction: Total knee arthroplasty (TKA) effectively relieves pain and improves functional capacity in end-stage knee osteoarthritis (OA). However, the influence of obesity, particularly as measured by body mass index (BMI), on TKA outcomes remains a subject of debate. This study aimed to investigate the relationship between BMI, knee subcutaneous fat measurements, and postoperative functional outcomes following TKA.

Methods: A retrospective analysis was conducted that included 100 patients (31 men and 69 women) who underwent primary TKA at a single university hospital. Patients were categorized based on BMI (kg/m^2^) into three groups: control (BMI <30), obese (BMI 30-39.9), and morbidly obese (BMI ≥40). Data collection included measurements of BMI, radiological fat indices, and assessment of joint function recovery using the 12-item Forgotten Joint Score (FJS-12) and flexion scores. Statistical analysis was performed to evaluate correlations and differences between BMI groups.

Results: The majority of patients were women (69%), with no significant BMI difference between genders. The surgical indication was predominantly OA, with a higher frequency seen in groups with BMI ≥30. Patients with a higher BMI underwent TKA at a younger age (p<0.00001). There was no significant difference in postoperative FJS-12 and flexion scores among BMI groups. Correlation analysis showed weak positive correlations between BMI/subcutaneous fat indices and functional outcomes. In addition, complication rates were low, with no reported infections.

Conclusion: Obesity did not independently affect TKA outcomes, with equivalent results observed across BMI groups. However, a higher BMI correlated with younger age at TKA. BMI and knee subcutaneous fat indices showed a poor predictive value for postoperative functional recovery. This study underscores the increasing proportion of obese patients undergoing TKA and the need for further research to understand the complex relationship between obesity, subcutaneous fat distribution, and TKA outcomes.

## Introduction

Osteoarthritis (OA) is a leading cause of disability worldwide, with the knee joint being the most commonly affected site. As the global population ages, the demand for total knee arthroplasty (TKA) has surged, making it one of the most frequently performed orthopedic procedures. The increasing frequency of TKA underscores its documented success, as evidenced in the literature [1-3]. Studies have shown a substantial rise in TKA procedures across different populations, particularly among individuals aged 70 to 79, as revealed by an analysis of Taiwan's National Health Insurance Research Database [1]. Notably, total knee replacement (TKR) rates are consistently higher in female patients, while hospitalization durations and associated expenses have notably decreased over time. Similar trends have been observed in other countries, including Australia, Denmark, Finland, Norway, and Sweden, where the rising incidence of knee replacements, particularly in women, highlights arthritis as an escalating public health concern [2,3]. Projections for countries like the UK and Germany further emphasize the necessity for proactive management strategies to address the increasing burden of OA and the subsequent demand for TKA [2,3].

While TKA is an effective treatment, understanding its postoperative outcomes remains critical due to the potential complications. Several studies have examined post-TKA issues such as patellar clunk or crepitus, which significantly impact patient recovery and satisfaction [4,5]. However, an important yet often debated factor influencing TKA outcomes is obesity.

Obesity, defined as a body mass index (BMI) > 30 kg/m², is a growing global concern, with projections indicating a significant increase in overweight and obese adults by 2030 [6]. The association between obesity and the necessity for TKA is well-documented, primarily due to increased joint loading and mechanical stress [7]. Research suggests that every kilogram of weight loss results in a fourfold reduction in knee joint load during daily activities, making obesity a crucial modifiable risk factor for knee OA [8-10]. Furthermore, obesity is linked to systemic metabolic disturbances that may complicate TKA efficacy and postoperative outcomes [8-11]. Several studies have reported that increased BMI correlates with heightened postoperative pain and complication rates, raising important questions about the long-term effectiveness of TKA in obese individuals [12,13]. However, the extent to which obesity impacts post-TKA recovery remains inconclusive. For instance, Boyce et al. [13] reported that body fat percentage (BFP) has limited predictive value for in-hospital outcomes or for assessing patient-reported pain, function, and health-related quality of life one year after surgery. Additionally, among non-morbidly obese patients, increasing BMI appears to have a minimal effect on TKA-related outcomes [13].

Beyond postoperative recovery, obesity has been associated with structural changes in the knee joint. Studies have shown that obese patients with knee OA exhibit narrower medial and lateral joint spaces compared to non-obese individuals, emphasizing the role of obesity in joint degeneration [14]. Furthermore, Ding et al. [15] highlighted that increasing BMI, especially in women, is strongly associated with knee cartilage defects and tibial bone enlargement, underscoring the importance of early intervention to mitigate OA progression in overweight and obese individuals.

While BMI remains the most widely used metric for evaluating obesity and predicting surgical outcomes, it does not account for fat distribution or localized adipose deposits that may influence postoperative recovery. As a result, the recent literature has shifted focus toward imaging-based fat indices that provide more anatomically relevant assessments. Measurements such as prepatellar and pretubercular fat thicknesses, quadriceps muscle content, and novel indices like the prepatellar fat thickness ratio (PFTR) and subcutaneous fat index have been explored as potential predictors of complications and functional outcomes following TKA. These localized parameters, assessed through MRI or CT, may offer improved specificity in evaluating risk, particularly regarding wound healing, joint function, and infection risk. Although not yet part of routine clinical assessment, these parameters underscore the growing recognition of body composition beyond BMI and may pave the way for more tailored preoperative risk stratification and postoperative management strategies [16-23].

The literature highlights the complex interplay between obesity and TKA outcomes. While obesity contributes to increased joint loading and systemic metabolic disturbances, it also necessitates individualized perioperative strategies to minimize complications [9-11]. Despite lower clinical scores in some measures, studies suggest that obese patients experience similar satisfaction and mid-term implant survival rates compared to non-obese individuals, supporting equitable access to TKA for this population [24]. Moreover, research indicates that preoperative weight loss and targeted BMI management may enhance surgical outcomes and reduce complication risks [25]. These findings collectively underscore the need for personalized, multifactorial approaches that account for individual patient characteristics to optimize TKA outcomes.

Building on these insights, this study aims to investigate the relationship between BMI, knee subcutaneous fat measurements, and postoperative functional outcomes following TKA. It is hypothesized that a higher BMI and increased preoperative soft tissue depths, specifically at the prepatellar, pretubercular, prequadriceps tendon, and medial regions, are inversely correlated with functional outcomes, complication rates, and patient satisfaction following total knee arthroplasty.

## Materials and methods

Study protocol

A total of 100 knee OA patients, including 31 male and 69 female patients, treated with TKA surgery at a single university hospital, King Hamad University Hospital (KHUH), from January to December 2017 were retrospectively enrolled in this study, following meeting the set inclusion and exclusion criteria. Data were collected from the radiology and health record system at the hospital, supplemented by communication with patients and their families. The collected data were then used to investigate the impacts of BMI, preoperative prepatellar, pretubercular, prequadriceps tendon, and/or medial soft tissue distances (soft tissue depths) with functional outcome, complications, and patient satisfaction after total knee arthroplasty. The study was approved by the Royal Medical Services Institutional Review Board Committee (RMS-KHUH/IRB/2024-781).

Inclusion/Exclusion Criteria

Patients who underwent primary total knee arthroplasty at the hospital during 2017, were less than 80 years of age at the time of the study and were willing to participate and able to verbally share their thoughts and opinions were included in the study. Conversely, patients were excluded if there was confirmed or suspected joint instability or prosthetic loosening, if they had sustained previous periprosthetic fractures, underwent revision procedures, or had constrained and mega prostheses. Patients were excluded from the study if they were deemed unsuitable for total knee replacement or had conditions that compromised their functional capacity for surgery, such as metabolic syndrome, immunocompromised status, or a high-risk American Society of Anesthesiologists (ASA) classification. The ASA Physical Status Classification System was used to assess preoperative fitness, with patients classified as ASA IV or higher, indicating severe systemic disease or moribund status, considered unfit for elective procedures like TKR. Additionally, individuals with severe comorbidities likely to affect postoperative recovery or functional outcomes were excluded. Cases with suboptimal preoperative radiographs, anatomical or degenerative deformities obscuring measurement landmarks, cognitive impairment, or incomplete data sets were also excluded.

Data collection methods, instruments, and measurements

Groups

Preoperative height and weight were collected, and BMI was calculated as BMI = Weight/Height^2^ (kg/m^2^). Patients were divided into three groups: patients with BMI <30 kg/m^2^ were included in the control group (n=24), patients with BMI 30-39.9 kg/m^2^ were included in the obese group (n=40), and patients with BMI ≥40 kg/m^2^ were included in the morbidly obese group (n=36).

Surgical Procedure

The patient was positioned supine before undergoing either general or spinal anesthesia. Utilizing the medial parapatellar approach, the surgical procedure involved rotating the patella outward to fully expose the knee joint, enabling comprehensive release of the soft tissue and correction of various knee deformities. The damaged surfaces of the distal femur and proximal tibia were then removed, followed by meticulous reconstruction of the joint surface to accommodate the chosen prosthetic joint, ensuring optimal bone bonding. Following thorough irrigation of the surgical site and hemostasis, the incision was carefully closed. After surgery, vigilant monitoring of vital signs was maintained, along with the judicious administration of antibiotics to prevent infection and intensified nutritional support to aid in the patient's recovery.

Evaluation of Joint Function Recovery

At the 24-month follow-up after total knee arthroplasty, patients completed the 12-item Forgotten Joint Score (FJS-12) questionnaire to assess the outcome quantitatively [26]. The scoring system was designed to reduce patient response burden and avoid cumbersome scores. The FJS-12 questionnaire evaluates participants' awareness of their artificial joints during activities of daily living (ADL). Each answer on the FJS-12 is scored on a scale from 1 to 5, with six response options available: never (1 point), almost never (2 points), sometimes (3 points), most of the time (4 points), all the time (5 points), or not applicable. These scores were totaled and converted to a percentage (ranging from 20% to 100%) to generate an overall score, with higher percentages indicating better outcomes. Additionally, immediate preoperative flexion and postoperative flexion at the two-year mark were recorded for each patient, and the difference in flexion scores was calculated.

Radiological Fat Index Measurements

Following soft tissue depth measurement (millimeter scale) obtained on direct pre-arthroplasty anterposterior (AP) and lateral radiographs, the following distances were recorded: (1) pretubercular distance (on the lateral x-ray view), determined from the most prominent point in the tibial tuberosity to the anterior skin line, perpendicular to the axis of the tibia; (2) prepatellar distance (on the lateral x-ray view), measured from the anterior cortex of the mid portion of the patella to the anterior skin line, perpendicular to the axis of the tibia; (3) prequadriceps tendon distance (on the lateral x-ray view), calculated as the distance between the shadow of the quadriceps tendon and the anterior skin line at a level 80 mm above the knee joint line and (4) medial joint-line-level soft tissue distance (on the AP x-ray view), measured from the medial-most point of the medial tibial plateau (excluding osteophytes) to the medial skin line at the level of the knee joint.

Statistical methods

Data analysis was performed using the Data Analysis ToolPak in Microsoft Excel (Microsoft Corporation, Redmond, USA). All data were collected on a Microsoft Excel spreadsheet. Results were analyzed, and are presented as means, percentages, and standard deviations for each BMI group as appropriate. Statistical significance was calculated in each study group (BMI <30/control, BMI 30-39.9/obese, BMI ≥40/morbidly obese) with significance set at p < 0.05.

Univariate subgroup analysis was performed in each category using the Pearson correlation coefficient test.

## Results

During the study period, 150 knee arthroplasties were performed. Of these, 100 patients had adequate follow-up data to allow for BMI calculation and outcome analysis. The majority of patients (n=69, 69%) were women, with no statistically significant differences between the median BMIs in men (36.4 ± 11.6) and women (36.8 ± 9.9) (p=0.73). The mean and median BMIs for both male and female patients can be seen in Table 1.

**Table 1:** BMI according to gender The BMI is expressed in kg/m². The mean BMI values have been represented as mean ± SD and the median BMI values have been represented as median ± IQR.

Mean BMI (kg/m^2^)		Median BMI (kg/m^2^)	
Male	Female	Male	Female
34.78 ± 6.91	37.46 ± 6.18	36.4 ± 11.6	36.8 ± 9.9

The main surgical indication for the primary knee arthroplasty was osteoarthritis; however, the frequency of OA was higher for the BMI ≥30 group versus the BMI <30 group (66% vs. 24%, respectively). The overall median age of male patients in the study was 67 ± 9.0 years: for the group with BMI <30, the median age was 67 ± 9.3 years (67-79); for the group with BMI 30-39.9, the median age was 67 ± 8.5 years (55-79) and for the group with BMI ≥40 the median age was 67 ± 9.0 years (60-75). Similarly, the overall median age of female patients in the study was 66 ± 9.0 years: for the group with a BMI <30, the median age was 67 ± 8.0 years (57-75); for the group with BMI 30-39.9, the median age was 65 ± 8.3 years (57-76) and for the group with BMI ≥40, the median age was 66.5 ± 9.0 years (49-76). The baseline characteristics of patients who underwent knee arthroplasties can be seen in Table 2.

**Table 2:** Baseline characteristics of patients who underwent knee arthroplasties Patients were divided into three groups based on their BMI, expressed in kg/m². The population count of each group has been represented as N (%), while the mean age has been expressed as mean ± SD, and the age range is represented in years.

	Population (N)		Mean age (years)		Age range (years)	
BMI (kg/m^2^)	Male	Female	Male	Female	Male	Female
Control group (<30)	11 (47.8%)	12 (52.2%)	69.5 ± 6.6	64.6 ± 6.3	67-79	57-75
Obese group (30-39.9)	12 (30.0%)	28 (70.0%)	67.0 ± 6.4	64.6 ± 6.2	55-79	57-76
Morbidly obese group (≥40)	7 (19.4%)	29 (80.6%)	66.3 ± 6.3	65.4 ± 6.5	60-75	49-76

**Figure 1:**
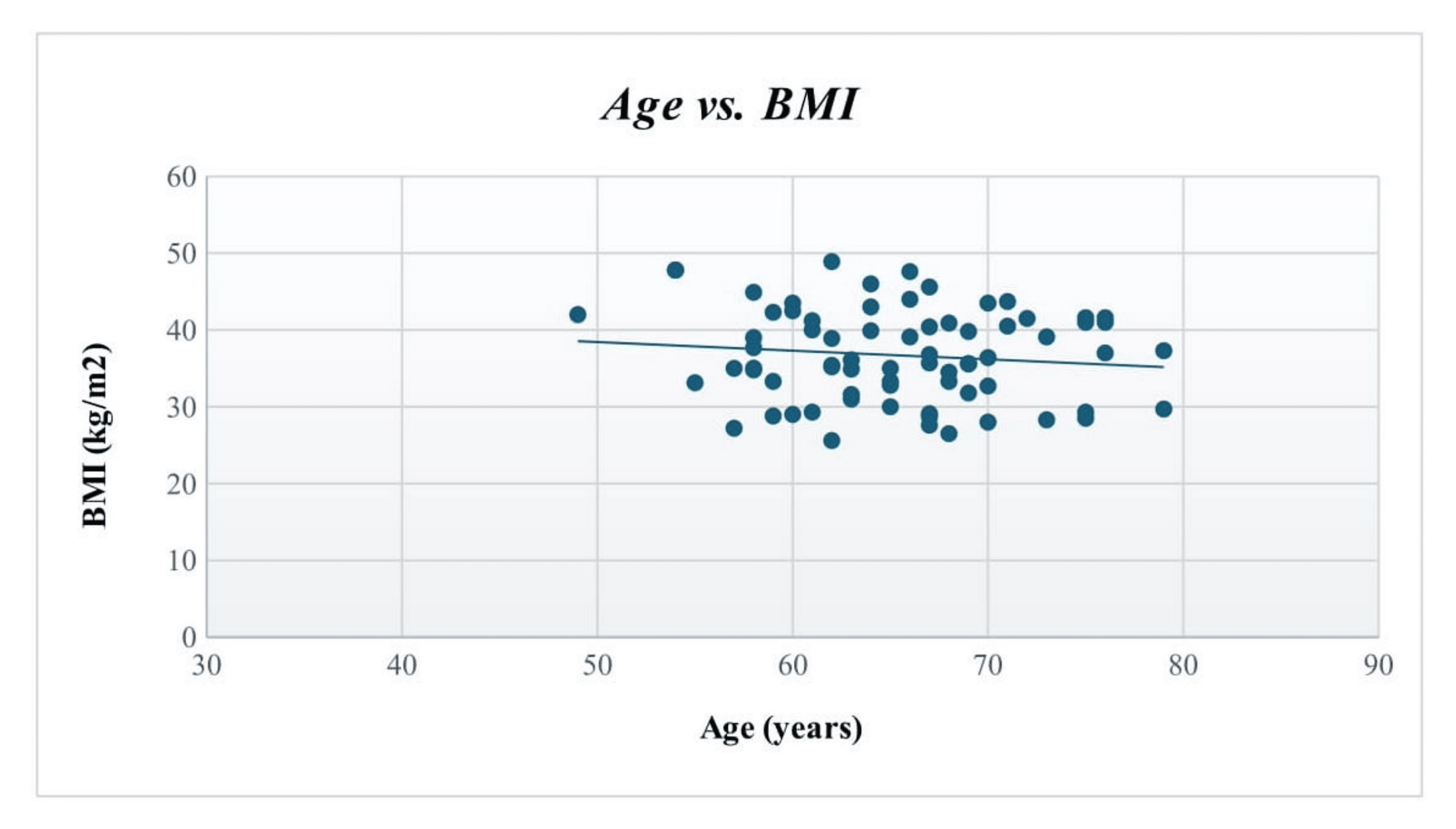
A scatter plot showing the correlation between BMI and age at which the knee arthroplasty surgery occurs Patients' individual BMI values are expressed in kg/m², while their respective age is expressed in years. The Pearson correlation coefficient (r) was used to calculate the p-value. A p-value less than 0.05 was considered significant.

A statistically significant trend (p<0.00001) was found for patients to receive knee arthroplasty at a younger age when they had a higher BMI. Figure 1 below displays a decreasing trend in BMI as age increases.

The mean values and ranges for all subcutaneous fat indices calculated for each category are shown in Tables 3, 4, respectively.

**Table 3:** Knee soft tissue depth measurements (means) for all groups The mean pretubercular, prepatellar, prequadriceps tendon, and medial joint-line-level soft tissue distances have all been expressed as mean ± SD.

	Mean (mm)			
BMI (kg/m^2^)	Pretubercular	Prepatellar	Prequadriceps	Medial
Control group (<30)	14.67 ± 7.99	9.46 ± 5.82	20.46 ± 8.79	33.39 ± 11.85
Obese group (30-39.9)	16.93 ± 7.88	9.59 ± 5.89	23.25 ± 8.97	42.79 ± 12.12
Morbidly obese group (≥40)	19.74 ± 7.92	12.70 ± 5.80	23.69 ± 8.82	37.93 ± 12.59

**Table 4:** Knee soft tissue depth measurements (ranges) for all groups

	Range (mm)			
BMI (kg/m^2^)	Pretubercular	Prepatellar	Prequadriceps	Medial
Control group (<30)	2.5-28.8	3.1-17.0	11.8-35.2	15.2-47.3
Obese group (30-39.9)	2.5-31.8	3.1-25.3	12.0-53.0	16.8-68.8
Morbidly obese group (≥40)	7.2-32.8	5.0-27.2	10.2-36.5	18.2-74.4

Relationship between knee functional recovery parameters after TKA in different BMI groups

**Figure 2:**
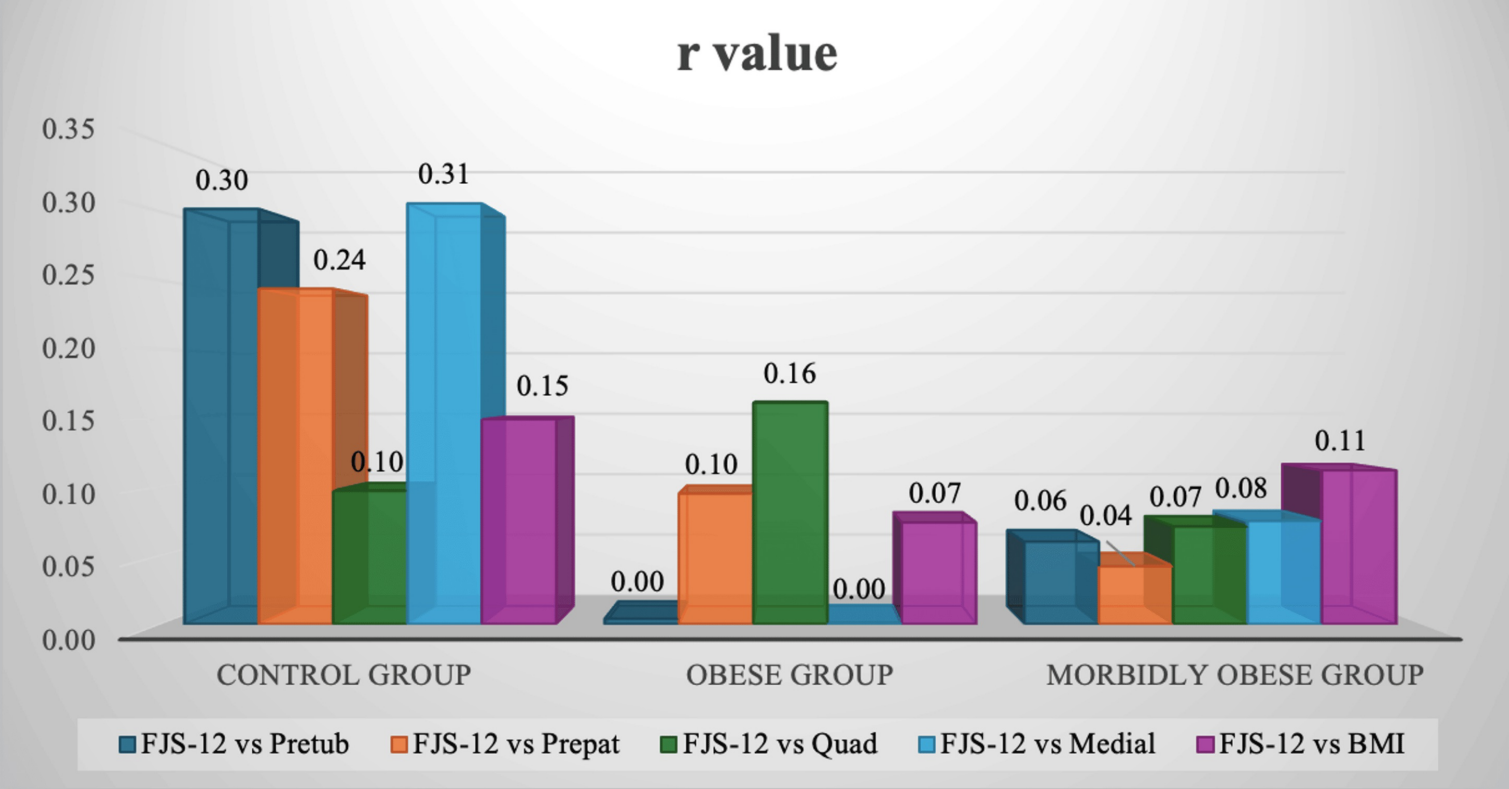
Pearson correlation coefficients (r) for postoperative knee functional outcomes using the 12-item Forgotten Joint Score (FJS-12) values compared to all preoperative subcutaneous fat indices in each BMI category The r values for each of the pretubercular, prepatellar, prequadriceps tendon, and medial joint-line-level soft tissue distances, preoperatively, compared to the FJS-12 values postoperatively in control, obese, and morbidly obese groups can be seen in the figure. The Pearson correlation coefficient was used to generate the r values: r values of 0.00-0.40 were considered weak, 0.4-0.6 were considered moderate, and 0.60-1.0 were considered strong.

**Figure 3:**
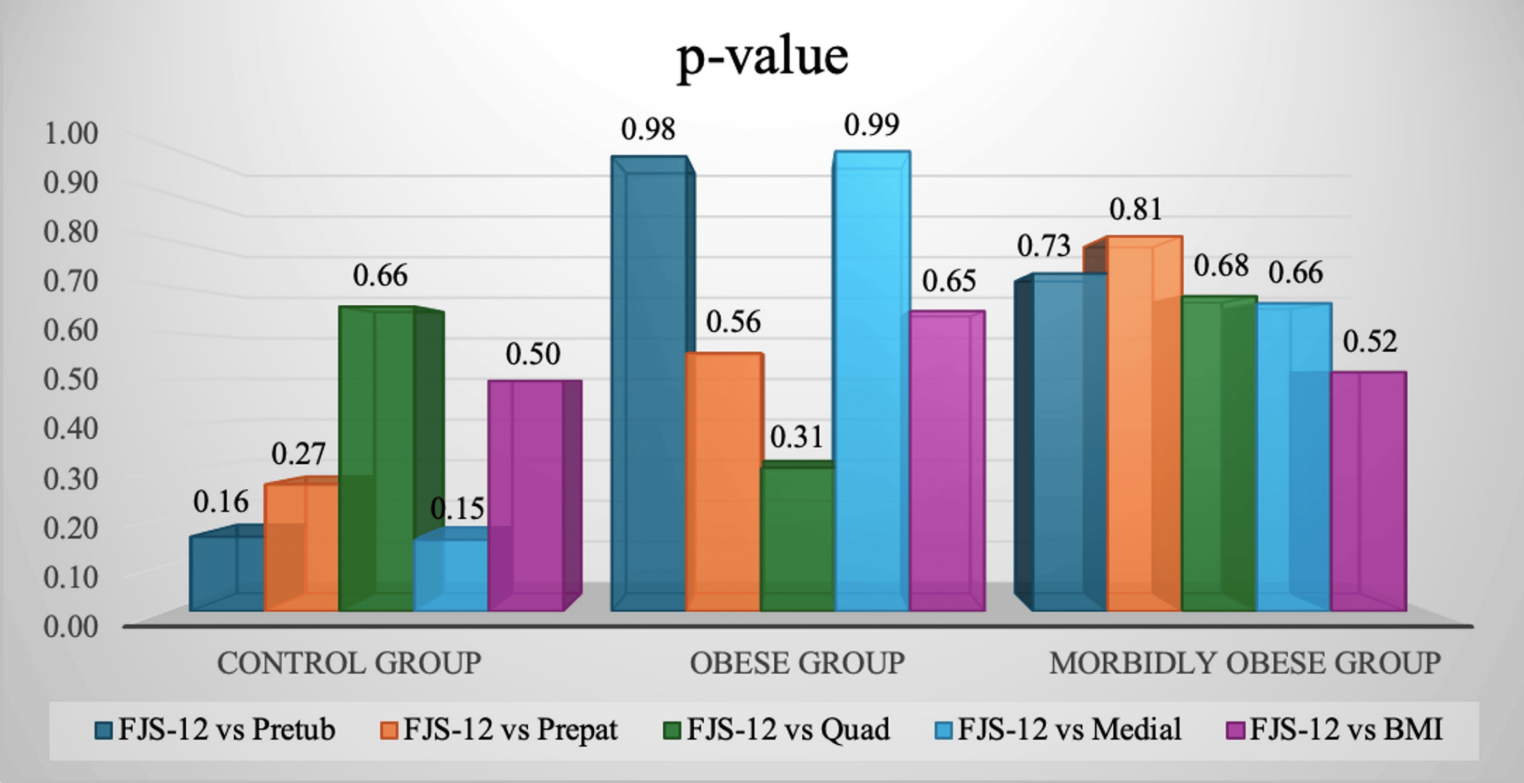
Probability values for postoperative knee functional outcomes using the 12-item Forgotten Joint Score (FJS-12) compared to all preoperative subcutaneous fat indices in each BMI category The p-values for each of the pretubercular, prepatellar, prequadriceps tendon, and medial joint-line-level soft tissue distances, preoperatively, compared to the FJS-12 values postoperatively in control, obese, and morbidly obese groups can be seen in the figure. The p-values were calculated by the Mann-Whitney U test of differences; p<0.05 was considered significant.

There was no significant difference in the postoperative 12-item FJS outcome scores among different BMI groups when comparing control with obese and morbidly obese categories (p=0.8181 and p=0.833, respectively). The median FJS-12 score was 53% in each category. Pearson correlation coefficient (r) relations were obtained for preoperative BMI and knee subcutaneous fat indices in each category with their postoperative FJS-12 values, which can be seen in Figure 2. Very weak positive r values were obtained. The majority of p-values were also significantly high (p>0.05), and are displayed in Figure 3. Thus, no statistical significance was found for BMI or any of the preoperative subcutaneous fat indices with respect to postoperative patient-reported outcome measures.

Relationship between postoperative flexion scores after TKA in different BMI groups

Passive range of motion (ROM) was assessed both preoperatively and postoperatively at patients' most recent follow-up visit that was greater than two years. There was no significant difference in the preoperative or postoperative ROM values among different BMI groups while comparing control with obese and control with morbidly obese categories (all p-values > 0.05) as shown in Table 5.

**Table 5:** p-values calculated using the Mann-Whitney U test of differences, for preoperative versus postoperative range of motion (ROM) across the BMI groups p<0.05 is considered significant.

Preoperative ROM		Postoperative ROM	
Control vs. obese	Control vs. morbidly obese	Control vs. obese	Control vs. morbidly obese
p = 0.435	p = 0.362	p = 0.23	p = 0.84

**Figure 4:**
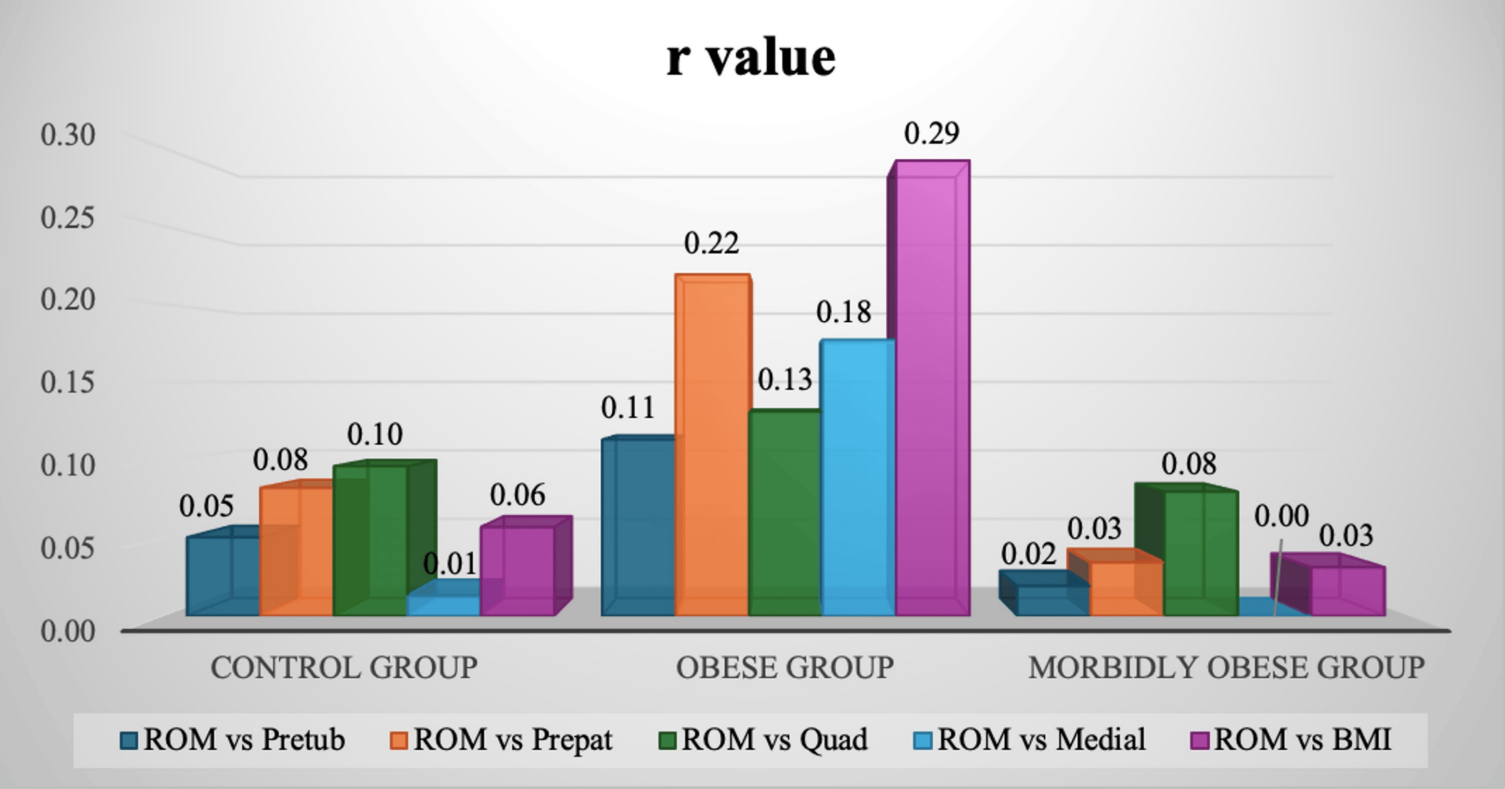
Pearson correlation coefficients (r) for postoperative flexion range of motion (ROM) scores compared to all preoperative subcutaneous fat indices in each BMI category The r values for each of the pretubercular, prepatellar, prequadriceps tendon, and medial joint-line-level soft tissue distances, preoperatively, compared to the flexion ROM postoperatively in control, obese, and morbidly obese groups can be seen in the figure. Pearson's correlation coefficient was used to generate the r values; r values of 0.00-0.40 were considered weak, 0.4-0.6 were considered moderate, and 0.60-1.0 were considered strong.

**Figure 5:**
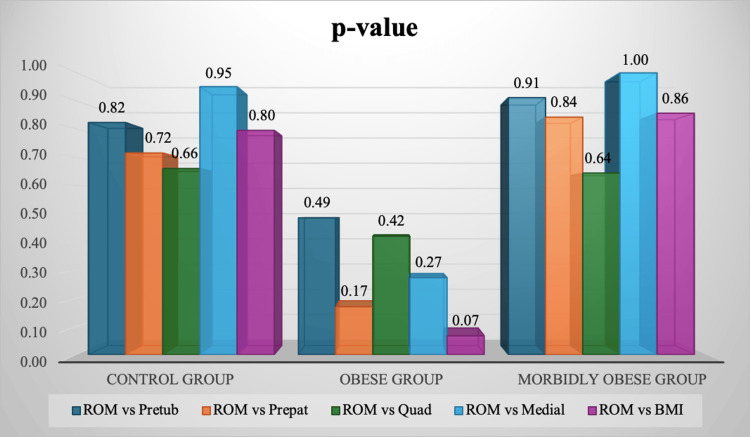
Probability values for postoperative flexion range of motion (ROM) scores compared to all preoperative subcutaneous fat indices in each BMI category The p-values for each of the pretubercular, prepatellar, prequadriceps tendon, and medial joint-line-level soft tissue distances, preoperatively, compared to the flexion ROM postoperatively in control, obese, and morbidly obese groups can be seen in the figure. The p-values were calculated by the Mann-Whitney U test of differences; p<0.05 was considered significant.

Pearson correlation coefficients were also obtained for preoperative BMI and knee subcutaneous fat indices in each category, along with their flexion scores differences, and are shown in Figure 4. Very weak positive r values were obtained. Most of the p-values were also significantly high (p>0.05) and can be seen in Figure 5. Thus, no statistical significance was found for BMI or any of the preoperative subcutaneous fat indices with respect to postoperative flexion scores.

## Discussion

On analyzing the demographic and clinical characteristics of our 100-patient knee arthroplasty cohort, where 69% (N=69) were women, our findings align with epidemiological trends indicating a higher prevalence of total knee replacement surgeries among female patients [1,2]. Notably, BMI analysis revealed no significant gender-based differences, with male patients exhibiting a mean BMI of 34.79 ± 6.21 kg/m² and female patients 37.46 ± 6.18 kg/m² [7,27]. This supports the existing literature highlighting elevated BMI as a key factor in the risk and progression of knee OA and the need for TKR [8,9,28].

Although our study did not find a direct association between BMI and postoperative outcomes, prior research emphasizes that increased BMI contributes to greater joint loading, which can exacerbate pain and lead to slow recovery [9,10,16]. Notably, every kilogram of weight loss is known to reduce knee joint load fourfold during daily activities, underscoring the value of weight management in knee OA patients [10,29].

OA was the primary indication for TKA in our cohort, with a notably higher incidence among patients with a BMI over 30, reinforcing the well-established link between obesity and knee OA [2,8]. However, this raises a crucial consideration: does a higher BMI necessarily translate to worse TKA outcomes? While obesity introduces challenges through increased joint loading and metabolic disturbances, our findings suggest that preoperative strategies, such as targeted weight loss and tailored surgical planning, mitigate these effects and help optimize outcomes.

Furthermore, our analysis revealed that higher BMI categories, especially in the morbidly obese group, were consistently associated with an increased knee soft tissue depth across most regions. This trend, in line with prior studies, reflects the anatomical influence of obesity, particularly, the contribution of subcutaneous fat to tissue thickness [12,13,30]. Importantly, we also observed increased variability in these measurements with rising BMI, underscoring the challenges this poses for surgical planning. Surgeons may need to account for such anatomical discrepancies to ensure optimal procedural outcomes across varying BMI profiles [7,9,31].

A consistent upward trend in mean soft tissue depth values was observed across BMI categories (control < obese < morbidly obese), accompanied by a parallel increase in standard deviations. This indicates not only higher average measurements in higher BMI groups but also greater variability. Such widening dispersion reflects the heterogeneity commonly seen within obese populations. Unlike individuals with a lower BMI, who tend to exhibit relatively uniform fat distribution and more consistent lifestyle patterns, those with a higher BMI may differ significantly due to factors such as central versus peripheral fat distribution, variations in muscle mass (e.g., sarcopenic vs. non-sarcopenic obesity), age, gender, and the presence of comorbidities like diabetes or prolonged corticosteroid use.

Despite our study finding no significant correlation between BMI or preoperative subcutaneous fat measurements and post-TKA functional outcomes, a growing body of literature emphasizes the role of localized fat distribution and muscle composition in predicting surgical risks and recovery. For instance, Watts et al. [16] highlighted that increased prepatellar and tubercular fat thicknesses are linked to early reoperations, reinforcing the clinical significance of site-specific fat deposits. Muscle and fat indices, particularly quadriceps muscle thickness and subcutaneous fat thickness, have also been shown to influence postoperative recovery, as greater muscle mass and lower echo intensity are associated with better outcomes [17]. Similarly, periarticular fat around the knee has been found to reduce wound complications, suggesting an optimal balance of fat distribution may be beneficial [18]. However, the complexity of predictive indices was evident in a study by Brown et al. [19], where the knee mass index (KMI) failed to predict the Oxford Knee Score, underscoring the limitations of novel metrics and the ongoing utility of traditional ones like BMI. This was echoed in another study by Ledford et al. [20], which demonstrated that percent body fat is a superior predictor of complications compared to BMI alone, highlighting the need for more precise compositional measurements. The introduction of the prepatellar fat thickness ratio in a study by Wagner et al. [21] offers a targeted tool that outperforms BMI in predicting surgical site infections, advocating for localized assessments in preoperative planning. Moreover, alterations in infrapatellar fat pad (IPFP) signal intensity have been linked with osteoarthritis progression and the likelihood of knee replacement, reflecting the role of fat-related inflammation in long-term joint outcomes [22]. Lastly, while the subcutaneous fat index correlates with obesity-related comorbidities, its limited predictive value in our findings suggests that subcutaneous fat, although relevant in broader obesity contexts, may not independently dictate post-TKA recovery [23]. Collectively, these studies support a nuanced approach to surgical prognostication -- one that incorporates fat distribution, muscle quality, and systemic inflammatory markers for a more holistic evaluation of patient outcomes.

An interesting finding in our data is the contrast between the reported median ages and the statistically significant trend in age at TKA across BMI groups. While patients with BMI 30-39.9 and ≥40 had slightly higher median ages compared to those with BMI <30, the overall trend showed that individuals with a higher BMI underwent TKA at a significantly younger age (p<0.00001). This apparent discrepancy raises important clinical considerations and may be influenced by both demographic and healthcare-related factors. One explanation is that obese patients often develop symptomatic knee OA earlier due to increased mechanical loading, prompting earlier surgical intervention. Additionally, younger individuals with a higher BMI may experience greater functional impairment and reduced quality of life, accelerating their path to surgery [14,32,33]. Evolving clinical practices and lower surgical thresholds for younger patients with significant disability may also contribute [14,34-36].

Conversely, the slightly higher median age observed in the high-BMI groups may reflect delays in surgical referral. Surgeons may defer TKA in obese patients due to perioperative risks, comorbidities, or the need for preoperative optimization. Socioeconomic barriers, prolonged conservative treatment, and disparities in healthcare access may also contribute to delayed intervention [14,34-36].

This apparent contradiction can be explained by the difference between the median age, which reflects central tendency, and the statistically significant trend, which captures the overall direction across the full sample. Although higher BMI is generally associated with younger age at surgery, the inclusion of several older morbidly obese patients broadened the age distribution and likely skewed the median age upward [32,33-37].

These findings underscore the complex interplay between obesity, age, and surgical decision-making in TKA and highlight the need for individualized evaluation that considers both metabolic and functional status [32,33-37].

Several studies support the notion that obese patients derive meaningful benefits from TKA, often reporting similar satisfaction levels and implant survival as non-obese patients despite lower absolute clinical scores [24,34,38,39]. For instance, one study found greater improvements in joint-specific outcomes in obese individuals, suggesting that TKA may offer substantial functional gains even in higher BMI populations. These findings argue against limiting surgical access based solely on BMI.

However, it is important to acknowledge the increased risk of complications such as restricted knee flexion, delayed wound healing, and infection in obese patients [29]. These challenges, often due to mechanical limitations from excess soft tissue, underscore the need for tailored perioperative management strategies. Thus, while BMI should not be used as a sole exclusion criterion, optimizing outcomes for obese TKA patients requires proactive risk mitigation and comprehensive preoperative planning.

Despite the complexities associated with obesity, this study reinforces that obese patients still experience substantial benefits from TKA, particularly in terms of pain relief and overall satisfaction. While some obese individuals may demonstrate modestly lower functional scores or encounter specific postoperative challenges, the mid-term survival rates and patient satisfaction are generally comparable to those of non-obese counterparts. These findings challenge the rationale for restricting access to TKA based solely on BMI and underscore the importance of adopting an individualized, holistic approach to surgical decision-making. Notably, our data also indicate that a higher BMI is associated with earlier age at surgery, suggesting a growing trend of younger obese patients requiring TKA, which has significant implications for healthcare planning and long-term joint management strategies.

Furthermore, this study highlights the need for proactive measures to address obesity-related joint deterioration before the need for surgery arises. Early lifestyle modifications, targeted physiotherapy, and emerging pharmacologic agents like GLP-1 receptor agonists (e.g., semaglutide, tirzepatide) offer promising avenues to delay or potentially prevent surgical interventions in at-risk populations [40]. While subcutaneous fat indices in this study showed limited predictive value for recovery outcomes, their association with BMI points to the broader biomechanical burden carried by obese knees. Future research should explore alternative markers of body composition and assess how integrated preoperative interventions, combining nutrition, physical therapy, and medical management, could optimize outcomes for obese patients undergoing TKA.

Limitations and strengths

While this study provides valuable insights into the role of BMI in TKA outcomes, certain limitations should be acknowledged. A progressive decrease in the long-term follow-up may have affected the generalizability of the results. Additionally, the retrospective nature of the study makes it susceptible to bias, and a prospective study design would allow for better control over these potential biases. The relatively small sample size may also limit the power of the study, making detecting statistically significant findings across all BMI categories difficult. Other confounding factors, such as the type of implant used, surgery timings, and medical comorbidities, were not considered, which could further influence postoperative outcomes.

Despite these limitations, the study provides important insights into the role of BMI and knee subcutaneous fat in TKA outcomes, particularly in a clinical setting where tight control over infection and surgical procedures has been achieved. The absence of wound infections in this study underscores the importance of stringent preoperative, intraoperative, and postoperative sterility protocols, which could mitigate complications in high-risk patients. The study's clinical strength lies in its real-world data, which reflects the outcomes of a hospital with robust safety measures.

Implications for clinical practice

This study suggests that while BMI alone does not independently predict TKA outcomes, it plays an essential role in identifying at-risk patients for complications. Notably, the correlation between higher BMI and younger age at TKA highlights the increasing number of obese patients undergoing surgery at a younger age. This demographic shift suggests that TKA may become a more common procedure in a population with an increasing prevalence of obesity. Therefore, future clinical guidelines may need to incorporate recommendations for early interventions to prevent or delay the need for TKA in obese patients, particularly those with other risk factors like joint degeneration and reduced mobility.

Recommendations

The findings of this study support the notion that BMI alone should not be used to restrict access to TKA for obese patients. Clinical decision-making should focus on a holistic approach that considers additional factors, such as the patient's functional status, comorbidities, and overall health. Future research should aim to establish optimal preoperative BMI thresholds for TKA and explore the role of body fat distribution in predicting postoperative recovery. Additionally, the integration of interdisciplinary approaches, including nutrition and physical therapy, could enhance preoperative preparation and improve long-term outcomes for obese patients undergoing TKA.

Future research directions

Future research should focus on developing a more comprehensive understanding of the role of obesity and fat distribution in post-TKA recovery. A prospective cohort study with a larger and more diverse sample would provide a clearer picture of the impact of BMI on long-term outcomes. Specifically, studies could focus on examining not only BMI but also other body composition measures, such as visceral fat or lean body mass, which may better predict recovery and complications following surgery. Furthermore, research could investigate how targeted preoperative interventions, such as weight loss programs or physical therapy, could improve outcomes for obese patients undergoing TKA.

The relationship between knee subcutaneous fat and postoperative recovery also warrants further exploration. In this study, knee subcutaneous fat indices showed poor predictive value, but understanding the underlying mechanisms may lead to better preoperative assessments and interventions. For example, patients with excess subcutaneous fat may benefit from a tailored rehabilitation program aimed at improving knee mobility and reducing post-surgical complications. This could help address the limitations in joint mobility observed in obese patients post-TKA.

## Conclusions

This study found no significant independent effect of BMI on TKA outcomes, with equivalent results observed across different BMI categories. However, BMI was correlated with younger age at surgery, suggesting that a higher proportion of younger, obese individuals are undergoing TKA. Furthermore, knee subcutaneous fat indices showed poor predictive value for postoperative recovery, highlighting the need for alternative measures of body composition in future research. Despite these findings, the study underscores the increasing number of obese patients undergoing TKA and the need for further research to fully understand the complex relationship between obesity, fat distribution, and functional recovery after surgery.
